# The Relationship Between Social-Emotional Learning Skills and Learning Motivation Among University Students: Exploring the Roles of Emotional Intelligence and Emotion Regulation

**DOI:** 10.3390/bs16091707

**Published:** 2026-09-21

**Authors:** Fatih Mutlu Özbilen, Alper Aytaç

**Affiliations:** 1 Çanakkale Vocational School of Social Sciences, Çanakkale Onsekiz Mart University, 17020 Çanakkale, Türkiye; fatihmutlu.ozbilen@comu.edu.tr; 2 Gürsu Science and Art Center, Ministry of National Education, 16580 Bursa, Türkiye

**Keywords:** university students, social-emotional learning skills, emotional intelligence, emotion regulation, learning motivation

## Abstract

This study examined the relationships among university students’ social-emotional learning (SEL) skills, emotional intelligence, emotion regulation, and learning motivation, with particular attention to a proposed serial indirect relationship through emotional intelligence and emotion regulation. A cross-sectional correlational design was employed with 372 students from the Faculties of Education and Arts and Sciences at a university in Türkiye, selected through stratified random cluster sampling. Data were analyzed using observed-variable path analysis in AMOS; the specific serial indirect effect in the original theoretical model was additionally estimated with PROCESS using bootstrap confidence intervals. SEL skills were positively related to learning motivation and emotional intelligence, emotional intelligence was positively related to emotion regulation, and emotion regulation was positively related to learning motivation. However, the direct relationships between SEL skills and emotion regulation and between emotional intelligence and learning motivation were not statistically significant. In the original theoretical model, the specific serial indirect relationship from SEL skills to learning motivation through emotional intelligence and emotion regulation was small and not statistically significant (β = 0.036, 95% CI [−0.0003, 0.0750]); therefore, the hypothesized serial indirect relationship was not supported. A post hoc parsimonious model excluding the two nonsignificant direct paths revealed a small but statistically significant serial indirect relationship (β = 0.043, 95% CI [0.005, 0.082]). This exploratory pattern should be interpreted cautiously and requires replication through longitudinal or experimental research. The findings may also inform curriculum developers by identifying social-emotional dimensions that warrant consideration in needs analysis and future curriculum-design and intervention studies in higher education, particularly in teacher education.

## 1. Introduction

Higher education represents an important developmental context in which students encounter increasingly complex academic, interpersonal, and emotional demands. During emerging adulthood, university students simultaneously engage in identity formation, career planning, and the development of interpersonal relationships (Arnett, 2000). Social and emotional capacities acquired and strengthened during this period have been linked to individuals’ long-term academic and professional development (Heckman & Kautz, 2012; Pascarella & Terenzini, 2005). Within this context, social-emotional learning (SEL) provides a broad framework for understanding the competencies that enable individuals to recognize and manage emotions, establish constructive relationships, understand others’ perspectives, and make responsible decisions (CASEL, 2020; Zins et al., 2004). The CASEL framework organizes these competencies into five interrelated domains: self-awareness, which involves recognizing one’s emotions and personal characteristics; self-management, which concerns regulating emotions and behaviors; social awareness, which involves understanding others’ perspectives and social contexts; relationship skills, which support constructive interpersonal interactions; and responsible decision-making, which involves making thoughtful choices in personal and social situations (CASEL, 2020). The conceptualization of SEL as a systematic framework draws on social-cognitive, emotional, and developmental perspectives (Greenberg et al., 2003; Weissberg et al., 2015), while contemporary approaches emphasize that these competencies develop through interactions among individuals and their educational, familial, and social environments (Osher et al., 2016). In the present study, the term SEL skills refers to the individual differences assessed by the study instrument, whereas SEL competencies refer more broadly to the domains conceptualized within the CASEL framework.

The educational relevance of SEL has been demonstrated in a substantial body of intervention research. Meta-analytic evidence indicates that school-based SEL programs are related to improvements in social-emotional skills, attitudes toward school, academic engagement, school functioning, peer relationships, and academic achievement (Cipriano et al., 2023; Durlak et al., 2011; Mahoney et al., 2018). Longitudinal findings further suggest that some positive outcomes linked to SEL interventions may persist beyond the implementation period (Taylor et al., 2017), while a recent systematic review and meta-analysis reported favorable academic outcomes among students participating in SEL programs (Ha et al., 2025). These findings indicate the broader educational relevance of SEL; however, intervention evidence should be distinguished from the focus of the present research. The current study does not evaluate the effectiveness of an SEL intervention. Instead, it examines individual differences in university students’ SEL skills and their statistical relationships with learning motivation and related emotional characteristics. Examining these relationships in higher education may be particularly valuable because university students are expected to manage academic responsibilities with increasing independence while simultaneously navigating changing social and emotional experiences.

One academic characteristic that may be particularly relevant to SEL skills is learning motivation, which refers to the willingness, effort, and persistence individuals display toward learning (Deci & Ryan, 2000; Pintrich, 2003). Self-determination theory provides an important framework for understanding this construct by conceptualizing motivation along a continuum of increasing self-determination. Amotivation reflects an absence of intentional motivation; external regulation concerns behavior driven primarily by external demands or rewards; introjected regulation involves internal pressures such as guilt or self-approval; identified regulation occurs when individuals personally value an activity; and integrated regulation reflects the incorporation of this value into the broader sense of self. Intrinsic motivation, in turn, refers to engagement arising from inherent interest and enjoyment (Deci & Ryan, 2000; Ryan & Deci, 2017). Autonomous forms of motivation are fostered when the basic psychological needs for autonomy, competence, and relatedness are satisfied and are related to adaptive learning, well-being, and academic persistence (Bureau et al., 2022; Niemiec & Ryan, 2009). Meta-analytic evidence similarly indicates that intrinsic and autonomous motivation have positive relationships with academic achievement and well-being, whereas amotivation is related to less favorable academic outcomes (Howard et al., 2021). Expectancy-value theory offers a complementary perspective by emphasizing students’ expectations of success and the value they assign to academic tasks (Wigfield & Eccles, 2000). In the present study, learning motivation was assessed using a measure comprising learning, performance-approach, and performance-avoidance goal orientations. From an achievement-goal perspective, learning goals concern developing competence and understanding, performance-approach goals concern demonstrating competence relative to others, and performance-avoidance goals concern avoiding the appearance of incompetence. These goal orientations may have different relationships with students’ engagement, persistence, and responses to academic challenges (Elliot & McGregor, 2001; Zenorini & Santos, 2010). SEL skills may be relevant to these motivational processes because emotional and social competencies are related to attitudes toward learning, self-efficacy, goal setting, and self-regulation (Bandura, 1997; Zimmerman, 2000; Zins et al., 2004). These theoretical connections provide the basis for expecting a positive relationship between SEL skills and learning motivation.

Emotional intelligence (EI) represents another construct that may help clarify the relationship between SEL skills and learning motivation. Within the ability model, emotional intelligence refers to the capacity to perceive emotions, use emotional information to facilitate thought, understand emotional meaning, and manage emotions (Mayer & Salovey, 1997; Mayer et al., 2016). These four branches represent increasingly complex forms of emotional information processing. Recent theoretical work further emphasizes that EI ability should be distinguished from emotionally intelligent behavior: possessing the capacity to process emotional information does not necessarily mean that this capacity will be enacted in everyday behavior, because its expression may also depend on motivational and contextual conditions (Levitats et al., 2025). Mixed models differ from the ability model by incorporating broader combinations of emotional abilities, personality-related characteristics, and social competencies. For example, Goleman’s (2020) approach emphasizes self-awareness, self-management, social awareness, and relationship management, whereas Bar-On’s (2006) emotional-social model incorporates personal and interpersonal competencies, adaptability, and stress management. Despite differences in conceptualization, these perspectives share an interest in the relationships of emotional information and competencies with adaptive functioning (Zeidner et al., 2009). In the present study, emotional intelligence was operationalized as self-reported emotional competencies involving the appraisal and regulation of one’s own and others’ emotions, rather than as performance on an ability-based emotional intelligence test. Emotional intelligence has shown a positive relationship with academic performance in meta-analytic research (MacCann et al., 2020) and has also been related to psychological well-being, self-efficacy, resilience, and perceived stress among university students (Gilar-Corbi et al., 2025; Ye et al., 2024). Previous research has further identified relationships between emotional intelligence and academic engagement and motivation (Brackett et al., 2011), coping with stress and anxiety (Salovey et al., 2002), and academic stress through social support (Ullah et al., 2023). Accordingly, students with stronger SEL skills may also report higher emotional intelligence, while emotional intelligence may be related to motivational processes in academic contexts.

A further construct relevant to this relational framework is emotion regulation, which concerns the processes through which individuals influence which emotions they experience, when these emotions occur, and how they are experienced or expressed (Gross, 2015). Gross’s process model distinguishes strategies according to when they operate within the emotion-generative process. Antecedent-focused strategies intervene before an emotional response is fully developed; cognitive reappraisal, for example, involves changing how an emotionally relevant situation is interpreted. Response-focused strategies operate after an emotional response has developed; expressive suppression involves inhibiting the outward expression of that response (Gross & John, 2003). A conceptually distinct classification concerns the relative adaptiveness of regulatory strategies. Strategies such as cognitive reappraisal, problem solving, and seeking social support are generally related to more adaptive functioning, whereas persistent suppression, rumination, and catastrophizing may be related to less favorable psychological outcomes (Aldao et al., 2010; Koole, 2009). In academic contexts, emotion regulation is closely related to how students respond to emotional experiences accompanying learning and achievement situations (Pekrun, 2006; Pekrun & Linnenbrink-Garcia, 2012). Students who regulate emotional experiences more adaptively may also demonstrate more constructive coping and motivational patterns when encountering academic difficulties (Pekrun et al., 2002). Recent research with university students has reported relationships between emotion regulation and academic motivation, approaches to learning, and academic performance (Rentzios et al., 2025; Wang & Wang, 2025). Adaptive and maladaptive regulatory strategies have shown different relationships with academic motivation under conditions of academic stress (Liang & Mao, 2025), while research on academic emotions highlights reciprocal relationships between emotional experiences and self-regulatory behavior during learning (Karagiannopoulou et al., 2023).

Although SEL skills, emotional intelligence, and emotion regulation all involve emotional functioning, they should not be treated as interchangeable constructs. SEL represents the broadest framework among the three because it incorporates emotional competencies together with explicitly social and interpersonal domains, including social awareness, relationship skills, and responsible decision-making (CASEL, 2020; Osher et al., 2016). Emotional intelligence has a more specific focus on the perception, understanding, use, and management of emotional information (Mayer & Salovey, 1997; Mayer et al., 2016). Accordingly, emotional intelligence overlaps primarily with the emotion-related components of SEL, whereas SEL additionally encompasses explicitly social, interpersonal, and decision-making competencies. Thus, although the two constructs share important conceptual features, emotional intelligence represents a more circumscribed emotional construct within the broader social-emotional domain addressed by SEL, and the two should therefore be regarded as conceptually related but non-equivalent. Emotion regulation, by comparison, concerns the processes and strategies through which emotional responses are modified or managed (Gross, 2015). Thus, these constructs overlap in their attention to emotional functioning but differ in scope and theoretical emphasis. SEL competencies may be related to greater emotional awareness and understanding (CASEL, 2020; Weissberg et al., 2015); emotional intelligence is theoretically related to individuals’ capacity to recognize and work with emotional information relevant to regulatory processes (Mayer & Salovey, 1997; Zeidner et al., 2009); and emotion regulation is related to motivational experiences in academic contexts (Karagiannopoulou et al., 2023; Pekrun & Linnenbrink-Garcia, 2012). These relationships provide a theoretical basis for examining whether emotional intelligence and emotion regulation form a sequential indirect relationship between SEL skills and learning motivation.

From a curriculum development perspective, examining these relationships may provide useful insights for higher education, particularly for teacher education curricula. University curricula are generally structured around disciplinary knowledge and academic competencies; however, students’ motivational engagement may also be related to social-emotional characteristics that shape how they respond to academic demands. This issue may be particularly relevant to pre-service teachers, who are preparing for a profession characterized by sustained interpersonal interaction, emotional demands, classroom relationship management, and responsibility for supporting students’ learning and development. Recent educational research also illustrates the broader professional relevance of emotional skills: perceived emotion regulation and emotional support in school leadership have demonstrated relationships with multiple dimensions of educator well-being (Floman et al., 2024). The findings of the present study cannot by themselves justify curricular intervention; however, they may inform future needs analyses and help identify social-emotional dimensions that warrant more systematic examination in the design and evaluation of teacher education curricula.

Despite growing research on each of these constructs, their relationships have rarely been examined together among university students within a single integrated model. Previous research has established relationships between SEL and academic outcomes, emotional intelligence and academic functioning, and emotion regulation and motivation; however, these lines of research have generally developed separately. This leaves uncertainty regarding whether the relationship between SEL skills and learning motivation includes a sequential indirect pattern involving emotional intelligence and emotion regulation. Addressing this issue may contribute to a more differentiated understanding of the relationships of closely related but conceptually distinct social-emotional characteristics with university students’ motivation to learn. Accordingly, the present study examined the proposed serial indirect relationship between SEL skills and learning motivation through emotional intelligence and emotion regulation among university students, together with the individual relationships specified in the proposed model.

Theoretical connections among SEL, attitudes toward learning, self-regulation, and adaptive forms of academic motivation provided the basis for H1 (Bureau et al., 2022; Zimmerman, 2000; Zins et al., 2004), while theoretical connections among SEL competencies, emotional awareness, and emotion management provided the basis for H2 and H3 (CASEL, 2020; Osher et al., 2016; Weissberg et al., 2015). Evidence concerning the relationships of emotional intelligence with emotion regulation and academic functioning supported H4 and H5 (Brackett et al., 2011; MacCann et al., 2020; Mayer & Salovey, 1997), and research demonstrating a relationship between emotion regulation and academic motivation supported H6 (Karagiannopoulou et al., 2023; Pekrun et al., 2002; Rentzios et al., 2025). Taken together, these theoretical relationships formed the basis for the serial indirect relationship proposed in H7.

**H1.**  *There is a positive relationship between university students’ social-emotional learning skills and learning motivation.*

**H2.**  *There is a positive relationship between university students’ social-emotional learning skills and emotional intelligence.*

**H3.**  *There is a positive relationship between university students’ social-emotional learning skills and emotion regulation.*

**H4.**  *There is a positive relationship between university students’ emotional intelligence and emotion regulation.*

**H5.**  *There is a positive relationship between university students’ emotional intelligence and learning motivation*.

**H6.**  *There is a positive relationship between university students’ emotion regulation and learning motivation.*

**H7.**  *There is a significant serial indirect relationship between university students’ social-emotional learning skills and learning motivation through emotional intelligence and emotion regulation, in that order.*

## 2. Method

This study employed a cross-sectional correlational survey design to examine the relationships among university students’ social-emotional learning skills, emotional intelligence, emotion regulation, and learning motivation. The hypothesized model specified a serial indirect relationship through emotional intelligence and emotion regulation. A correlational survey design is a quantitative research design that aims to identify patterns of covariation and relational structures among two or more variables (Karasar, 2012). This design enables the examination of naturally occurring relationships among variables without introducing an intervention or experimental condition. It is therefore widely used in the social and behavioral sciences to describe the relationships between individuals’ psychological characteristics and academic processes (Creswell & Creswell, 2018).

### 2.1. Participants

The study population consisted of students enrolled in the faculties of education and arts and sciences at a university in Türkiye during the 2025–2026 academic year. The sample consisted of 372 university students who were enrolled in class sections selected through stratified random cluster sampling and voluntarily agreed to participate in the study. Stratified random cluster sampling is an approach in which the population is divided into strata according to specified characteristics, natural clusters within each stratum are treated as sampling units, and clusters are randomly selected from each stratum (Creswell & Creswell, 2018; Karasar, 2012). This method is considered a functional sampling strategy in educational research because it supports the representation of subgroups such as faculties and year levels while facilitating data collection through natural clusters such as class sections.

In the present study, eight strata were first formed based on the two faculties and four year levels. The class sections within each stratum were then identified, and each section was treated as a natural cluster and primary sampling unit. One class section was randomly selected from each stratum, resulting in a total of eight class-section clusters. All students enrolled in the selected sections were invited to participate, and no second-stage sampling was conducted at the individual student level within the selected sections. The study initially aimed to reach approximately 400 students. However, because participation was voluntary, the analyses were conducted using data obtained from 372 students who agreed to participate and provided valid responses. Differences in the numbers of participants across faculties and year levels reflected both the numbers of students enrolled in the selected sections and the levels of voluntary participation.

Of the participating students, 198 were enrolled in the Faculty of Education and 174 in the Faculty of Arts and Sciences. The sample comprised 277 women and 95 men. Regarding year level, 75 participants were first-year students, 138 were second-year students, 56 were third-year students, and 103 were fourth-year students.

### 2.2. Instruments

#### 2.2.1. Social-Emotional Learning Skills Scale

The Social-Emotional Learning Skills Scale was developed by Kabakçı and Korkut Owen (2010) to assess the social-emotional learning skills of students in Grades 6–8. The scale-development study was conducted with 1343 students attending various elementary schools. Participants were divided into separate groups for the construct validity, test–retest reliability, and internal consistency analyses.

The instrument is a four-point Likert-type scale consisting of 40 items and four subscales: Problem-Solving Skills (11 items), Skills That Enhance Self-Worth (10 items), Coping with Stress Skills (10 items), and Communication Skills (9 items). Responses are rated from 1 (“Not at all appropriate for me”) to 4 (“Completely appropriate for me”). In the exploratory factor analysis (EFA), the Kaiser–Meyer–Olkin value was 0.89, and a four-factor structure was obtained. The first confirmatory factor analysis (CFA) indicated that the model-fit indices were within acceptable ranges (CMIN/DF = 1.76, RMSEA = 0.03, GFI = 0.90, CFI = 0.96, AGFI = 0.89, NFI = 0.92, NNFI = 0.96, and SRMR = 0.04). A second CFA conducted using a different dataset also produced model-fit indices within acceptable ranges. Cronbach’s alpha coefficients ranged from 0.61 to 0.83 across the subscales and were 0.88 for the total scale. The test–retest reliability coefficient for the total score was 0.85. In the present study, Cronbach’s alpha and McDonald’s omega coefficients for the total scale were 0.91 and 0.90, respectively.

Kocakülah and Kırtak Ad (2015) examined whether this scale, which was originally developed for middle school students, could be used with university students. In their study involving 514 university students, CFA was conducted, and the model-fit indices were found to be within acceptable ranges (CMIN/DF = 2.70, RMSEA = 0.05, CFI = 0.93, GFI = 0.84, NFI = 0.89, NNFI = 0.92, and AGFI = 0.82). The scale was also used with a university sample in Türkiye in the study conducted by Canlı et al. (2025). These findings support the use of the scale with university students.

Examples of items from the scale include:

“When I have a problem, I first try to understand what the problem is.”

“I can select and apply the most appropriate solution among the solutions I have identified for my problem.”

“When making a decision, I try to anticipate the positive and negative consequences of each option.”

“When speaking, I support what I say through my facial expressions.”

“I can express my wishes and needs comfortably.”

#### 2.2.2. Rotterdam Emotional Intelligence Scale

The Rotterdam Emotional Intelligence Scale was developed by Pekaar et al. (2018) and adapted into Turkish by Tanrıöğen and Türker (2019). The adaptation study was conducted with 487 teachers. The scale was first translated into Turkish, and linguistic validity analyses were performed. A pilot administration was subsequently conducted to prepare the instrument for implementation. EFA was performed using data obtained from 240 participants, whereas CFA was conducted using data from the remaining 247 participants. The Kaiser–Meyer–Olkin value obtained in the EFA was 0.94.

The scale consists of 28 items and four subscales: Appraisal of One’s Own Emotions (7 items), Appraisal of Others’ Emotions (7 items), Regulation of One’s Own Emotions (7 items), and Regulation of Others’ Emotions (7 items). It is a five-point Likert-type scale, with responses ranging from “Strongly disagree” to “Strongly agree.” The CFA results indicated acceptable model fit (CMIN/DF = 2.64, SRMR = 0.05, GFI = 0.87, AGFI = 0.85, IFI = 0.94, TLI = 0.93, CFI = 0.94, and RMSEA = 0.05). Cronbach’s alpha coefficients ranged from 0.89 to 0.93 across the subscales and were 0.94 for the total scale. In the present study, Cronbach’s alpha and McDonald’s omega coefficients for the total scale were 0.94 and 0.93, respectively.

Examples of items from the scale include:

“I am always aware of how I feel.”

“I am aware of the emotions of the people around me.”

“I can control my own emotions.”

“Even when I am angry, I can remain calm.”

“I can make another person feel differently.”

#### 2.2.3. Emotion Regulation Questionnaire

The Emotion Regulation Questionnaire was developed by Gross and John (2003) and adapted into Turkish by Eldeleklioğlu and Eroğlu (2015). During the adaptation process, data were first collected from 30 participants to examine linguistic equivalence. Validity and reliability analyses were then conducted using data obtained from 442 university students enrolled in different faculties of a university. The test–retest study was conducted with 90 students at a three-week interval.

The scale consists of 10 items and two subscales: Reappraisal (6 items) and Suppression (4 items). It is a seven-point Likert-type scale, with responses ranging from 1 (“Strongly disagree”) to 7 (“Strongly agree”). In the present study, the overall ERQ score was used as an operational indicator of participants’ self-reported emotion-regulation responding rather than as evidence that cognitive reappraisal and expressive suppression constitute a single underlying strategy. This analytical decision was consistent with the purpose of the hypothesized model, which focused on the broader role of emotion regulation in the proposed relational structure rather than on strategy-specific pathways. Demirhan and Solmaz (2024) similarly treated the 10-item ERQ as a single emotion-regulation variable in their analyses rather than reporting cognitive reappraisal and expressive suppression as separate variables; emotion regulation was represented by a single overall descriptive score and entered as a single moderator in their path model. Following this analytical approach, the mean score across all 10 ERQ items was used in the present study. EFA was not conducted in the Turkish adaptation study; instead, the theoretically specified two-factor structure was tested using CFA. The CFA results indicated good model fit (CMIN/DF = 1.94, CFI = 0.98, RMSEA = 0.04, and GFI = 0.99). Cronbach’s alpha coefficients were 0.78 for Reappraisal and 0.73 for Suppression. The corresponding test–retest reliability coefficients were 0.74 for Reappraisal and 0.72 for Suppression. In the present study, Cronbach’s alpha and McDonald’s omega coefficients for the total scale were 0.84 and 0.83, respectively. In addition, a one-factor CFA conducted using the data from the present study was used to examine the empirical plausibility of representing the 10 ERQ items with an overall composite score. The model demonstrated acceptable fit (CMIN/DF = 3.68, CFI = 0.94, GFI = 0.94, IFI = 0.94, NFI = 0.92, TLI = 0.90, RMSEA = 0.08, and SRMR = 0.05). These findings provided additional empirical support for the use of the overall ERQ score as an operational composite in the present analyses, while not implying that cognitive reappraisal and expressive suppression are conceptually identical or constitute a single emotion-regulation strategy.

Examples of items from the scale include:

“When I want to feel more positive emotions, such as joy or amusement, I change what I am thinking about.”

“When I want to feel fewer negative emotions, such as sadness or anger, I change what I am thinking about.”

“When I am faced with a stressful situation, I make myself think about it in a way that helps me remain calm.”

“I keep my emotions to myself.”

“When I am experiencing positive emotions, I am careful not to express them.”

#### 2.2.4. Motivation for Learning in Higher Education Scale

Motivation for Learning in Higher Education Scale was developed by Zenorini and Santos (2010) and adapted into Turkish by Erduran Tekin (2023). The adaptation study was conducted with a total of 1325 university students. Linguistic validity was examined with 37 students, criterion-related validity with 63 students, and reliability with 31 students. EFA was conducted using data from 741 students, whereas CFA was conducted using a separate sample of 453 students to independently evaluate the factor structure.

The Turkish form is a three-point Likert-type scale with the response options “Disagree” (1), “Undecided” (2), and “Agree” (3). The EFA yielded a 23-item, three-factor structure. The subscales were Performance-Avoidance Goals (7 items), Learning Goals (8 items), and Performance-Approach Goals (8 items). The Kaiser–Meyer–Olkin value obtained in the EFA was 0.86. The CFA results indicated acceptable model fit (CMIN/DF = 2.24, RMSEA = 0.05, CFI = 0.90, GFI = 0.91, AGFI = 0.90, and SRMR = 0.06). Cronbach’s alpha coefficients ranged from 0.82 to 0.87 in the EFA sample and from 0.79 to 0.82 in the CFA sample. In the present study, Cronbach’s alpha and McDonald’s omega coefficients for the total scale were 0.83 and 0.84, respectively. Although the scale comprises three conceptually distinct goal-orientation dimensions, the overall score was used in the present study as an operational composite indicator of learning motivation. This scoring approach is consistent with previous studies using the same Turkish version of the scale, in which learning motivation has been represented by an overall score in correlational, regression, and pretest–posttest analyses (Çevik & Demirdağ, 2026; Oner et al., 2026; Yıldırım, 2026). Notably, Oner et al. (2026) examined both the overall EMAPRE-U score and its individual subscale scores, demonstrating the analytical use of the total score alongside the scale’s distinct goal-orientation dimensions. The satisfactory internal consistency of the overall score in the present sample provides additional support for this analytical use. Nevertheless, the overall score should be interpreted as a broad operational indicator of learning motivation rather than as evidence that learning, performance-approach, and performance-avoidance goal orientations constitute a single unidimensional construct.

Examples of items from the scale include:

“I engage in my academic work because I am interested in it.”

“I enjoy it when a topic encourages me to learn more.”

“I want to stand out from the other students in my class.”

“I enjoy completing assignments that make me think.”

“One reason I do not participate in class is to avoid appearing uninformed.”

### 2.3. Procedure

Before the study commenced, ethical approval was obtained from the Çanakkale Onsekiz Mart University Social Sciences and Humanities Ethics Committee (protocol code 2026-YÖNP-0288; date of approval: 17 March 2026). The study was conducted in accordance with the ethical principles of the Declaration of Helsinki. Following ethical approval, the implementation plan was developed. As part of the stratified random cluster-sampling procedure, the class sections at each year level in the two faculties were identified, and each section was treated as a natural cluster. One class section was randomly selected within each stratum, and all students enrolled in the selected sections were invited to participate in the study.

Before data collection, participants were informed about the purpose and scope of the study and the data-collection process. They were informed that participation was entirely voluntary, that declining to participate would not result in any adverse consequences, and that they could withdraw from the study at any time without providing a reason. Informed voluntary consent was obtained from all participants. Participants were also assured that their responses would remain confidential and would not be linked to personally identifiable information. Data were collected online over a 12-day period in April 2026. The collected data were used solely for scientific research purposes and stored in a manner designed to prevent unauthorized access. The study initially aimed to reach approximately 400 students. Because participation was voluntary, the analyses were conducted using data obtained from 372 students.

### 2.4. Data Analysis

Before the main analyses, the distributional characteristics of the dataset were examined. The dataset contained no missing data. The univariate distributions of the mean scores for each scale were assessed using skewness and kurtosis coefficients together with the Shapiro–Wilk and Kolmogorov–Smirnov tests. Because significance-based normality tests may be sensitive to minor distributional deviations in relatively large samples, their results were interpreted together with the skewness and kurtosis coefficients. Multivariate normality was examined using Mardia’s multivariate kurtosis coefficient and its critical ratio. The results of the normality analyses are presented in the Section 3.

Because participants were nested within the selected class sections, possible intraclass dependence was assessed before the main analyses. ICC(1) coefficients were estimated for social-emotional learning skills, emotional intelligence, emotion regulation, and learning motivation using a one-way random-effects model, with class section as the grouping variable. These coefficients were used to evaluate the extent of within-cluster dependence across the eight sampled class-section clusters.

Because the self-report measures were administered to the same participants during the same data-collection process, the possibility of common method variance was also evaluated. Harman’s single-factor test was performed for this purpose, and all items were entered simultaneously into an unrotated exploratory factor analysis (Harman, 1976; Podsakoff et al., 2003). The first factor accounted for 21% of the total variance. The fact that a single factor did not account for a substantial proportion of the total variance indicated that common method variance was not a dominant concern in the present study.

Contemporary mediation approaches were adopted to test the proposed serial indirect relationships. Accordingly, indirect relationships were not evaluated solely through the traditional causal-steps approach based on the significance of the total or direct relationship; instead, the direct assessment of the indirect effect was treated as the primary criterion. Zhao et al. (2010) stated that the significance of the indirect effect constitutes the principal criterion in mediation analysis and that traditional interpretations based on the distinction between full and partial mediation have important limitations. Similarly, Hayes (2022) recommended evaluating indirect effects through bootstrap confidence intervals and not treating the significance of the total effect as a necessary prerequisite for mediation. 

Observed-variable path analysis was conducted using IBM SPSS Amos, version 21.0 (IBM Corp., Armonk, NY, USA) to test the proposed model. Path analysis is a structural analytic technique that allows multiple direct and indirect relationships among variables to be modeled and tested simultaneously (Kline, 2023). Each construct was represented by the mean score obtained from the corresponding scale. This observed-variable approach enabled the direct relationships and serial indirect paths among the variables to be tested within the same model (Hair et al., 2014).

The total relationship between SEL skills and learning motivation was first tested to evaluate H1. The initial theoretical serial model was then estimated to test the direct paths proposed under H2–H6 and the total indirect relationship across all indirect paths. After the paths corresponding to H3 and H5 were not statistically supported, a more parsimonious model excluding these two paths was also evaluated. Because this second model was developed after examining the results of the initial model, it was treated as exploratory rather than confirmatory. The initial serial model included all possible direct paths and was therefore just-identified. Consequently, model-fit indices were not interpreted for the initial model. Model fit was assessed using the exploratory parsimonious model, which had positive degrees of freedom.

**Figure 1:**
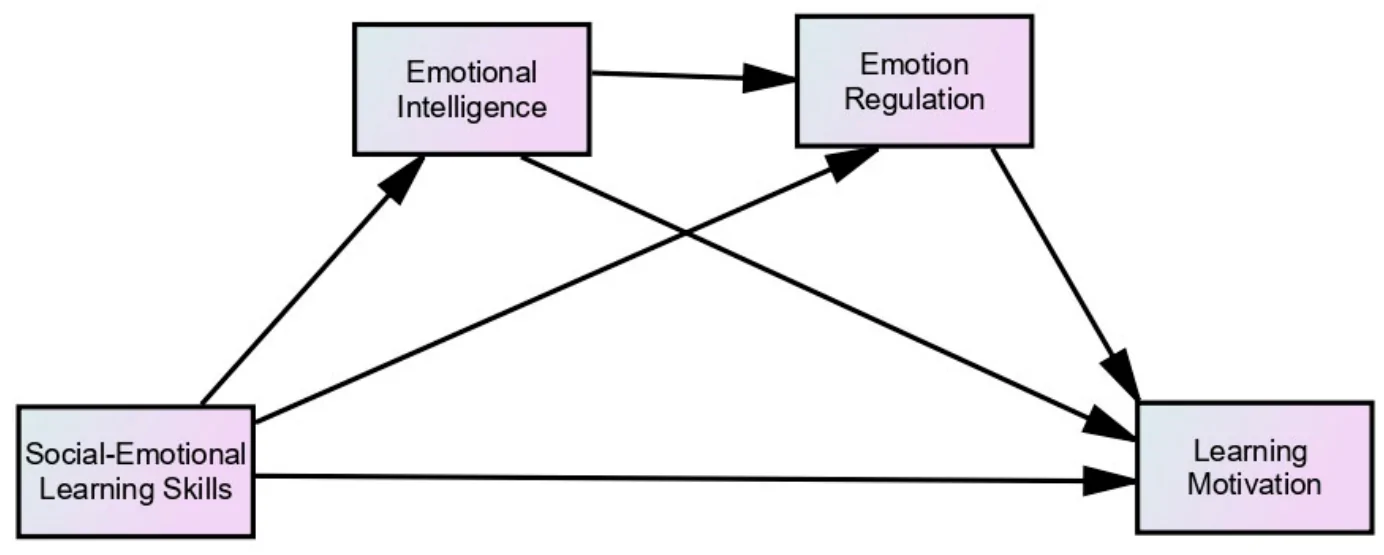
Initial theoretical serial indirect model.

The statistical significance of the total indirect relationship in the initial theoretical model and the serial indirect relationship in the exploratory parsimonious model was evaluated in AMOS using 95% bootstrap confidence intervals based on 5000 resamples. In addition, to estimate the specific serial indirect relationship from SEL skills to learning motivation through emotional intelligence and emotion regulation within the complete original theoretical model, a path-specific analysis was conducted using PROCESS Macro, version 5.0 (Andrew F. Hayes, Calgary, AB, Canada), for IBM SPSS Statistics, version 27.0 (IBM Corp., Armonk, NY, USA), following the serial mediation procedure described by Hayes (2022). For this additional analysis, the completely standardized specific indirect effect and its 95% bootstrap confidence interval were estimated using 20,000 bootstrap resamples. An indirect relationship was considered statistically significant when its confidence interval did not include zero. The bootstrap approach enables confidence intervals to be estimated without requiring the sampling distribution of the indirect effect to be normally distributed (Preacher & Hayes, 2008). A significance level of 0.05 was used to evaluate the direct paths. The fit of the exploratory parsimonious model was evaluated using CMIN/DF, RMR, SRMR, GFI, NFI, and RFI. The initial theoretical serial indirect model tested in the study is presented in Figure 1.

## 3. Results

Before presenting the main findings, the descriptive statistics for the study variables were examined. The relevant results are presented in Table 1.

Table 1 presents the descriptive statistics and univariate normality results for the study variables. The skewness coefficients ranged from −0.28 to 0.06, whereas the kurtosis coefficients ranged from −0.22 to 0.42; thus, all values were within the ±1 range. The Shapiro–Wilk test indicated statistically significant deviations from normality for social-emotional learning skills, emotional intelligence, and emotion regulation scores, whereas the Kolmogorov–Smirnov test indicated statistically significant deviations for emotion regulation and learning motivation scores. However, significance-based normality tests assess exact normality and may be highly sensitive to minor distributional deviations in relatively large samples. Therefore, particularly in large samples, normality should not be evaluated solely on the basis of the significance levels of formal tests; indices reflecting the shape and magnitude of distributional deviations, such as skewness and kurtosis coefficients, should also be considered (Ghasemi & Zahediasl, 2012; Kim, 2013; Shatz, 2024).

It was also considered that the scores used in the study were mean scores obtained from multi-item Likert-type scales. Although responses to individual Likert-type items are discrete and bounded, parametric analyses conducted using total or mean scores from multi-item scales have been reported to be robust against moderate deviations from normality (Finney & DiStefano, 2013; Norman, 2010). The examination of multivariate normality yielded Mardia’s multivariate kurtosis coefficient of 1.648, with a critical ratio of 2.334. When the formal test results, univariate skewness and kurtosis coefficients, and multivariate kurtosis indicator were considered together, no substantial distributional problem that would preclude the planned path analysis was identified.

Possible intraclass dependence across the eight class-section clusters was also examined before the main analyses. The ICC(1) estimates were −0.003 for social-emotional learning skills, 0.019 for emotional intelligence, 0.009 for emotion regulation, and 0.002 for learning motivation. The negative ICC estimate for social-emotional learning skills indicates essentially no between-cluster dependence and was therefore interpreted as zero. Overall, the ICC values were negligible to very small, indicating limited intraclass dependence across the sampled class sections.

Bivariate correlations among social-emotional learning skills, emotional intelligence, emotion regulation, and learning motivation were examined before the path analysis. The results are presented in Table 2.

As shown in Table 2, all principal study variables were positively and statistically significantly correlated. The strongest association was observed between social-emotional learning skills and emotional intelligence (r = 0.67), followed by the relationship between emotional intelligence and emotion regulation (r = 0.47). Social-emotional learning skills were also positively related to emotion regulation (r = 0.35) and learning motivation (r = 0.25), while emotional intelligence (r = 0.22) and emotion regulation (r = 0.20) were positively related to learning motivation. Using the conventional interpretive ranges summarized by Schober et al. (2018), the association between SEL skills and emotional intelligence can be regarded as moderate. Thus, although these two constructs were meaningfully related, the observed association was consistent with their treatment as conceptually related but distinguishable constructs.

The findings are presented in the order of the hypotheses. Table 3 presents the total relationship and direct path coefficients corresponding to the study hypotheses. Panel A shows the total relationship between social-emotional learning skills and learning motivation examined under H1. Panel B presents the direct path coefficients estimated in the initial theoretical serial indirect model.

Table 3 presents the path coefficients for the total relationship model and the initial theoretical serial indirect model. As shown in Panel A, the total relationship between social-emotional learning skills and learning motivation was positive and statistically significant (β = 0.254, CR = 5.062, *p* < 0.001). Accordingly, H_1_ was supported.

The direct paths in the initial theoretical serial indirect model are presented in Panel B. A positive and statistically significant relationship was found between social-emotional learning skills and emotional intelligence (β = 0.676, CR = 17.687, *p* < 0.001); therefore, H_2_ was supported. In contrast, the direct relationship between social-emotional learning skills and emotion regulation was not statistically significant (β = 0.064, CR = 1.042, *p* = 0.298); therefore, H_3_ was not supported.

A positive and statistically significant relationship was found between emotional intelligence and emotion regulation (β = 0.476, CR = 10.439, *p* < 0.001), supporting H_4_. The direct relationship between emotional intelligence and learning motivation was not statistically significant (β = 0.043, CR = 0.596, *p* = 0.551); therefore, H_5_ was not supported. The direct relationship between emotion regulation and learning motivation was positive and statistically significant (β = 0.133, CR = 2.528, *p* < 0.05), supporting H_6_.

When the mediating variables were included in the model, the direct relationship between social-emotional learning skills and learning motivation remained statistically significant (β = 0.182, CR = 2.687, *p* < 0.05). However, this finding alone does not demonstrate that the indirect relationship was statistically significant. The bootstrap results for the total indirect relationship and the specific serial indirect relationship in the initial theoretical model are presented in Table 4.

Table 4 presents both the total indirect relationship across all indirect paths and the specific serial indirect relationship corresponding to the hypothesized sequence in the complete original theoretical model. The total indirect relationship between social-emotional learning skills and learning motivation across all indirect paths was not statistically significant (β = 0.072, 95% CI [−0.033, 0.176]). More importantly, the specific serial indirect relationship from social-emotional learning skills to learning motivation through emotional intelligence and emotion regulation was also small and not statistically significant (β = 0.036, 95% CI [−0.0003, 0.0750]). Because the bootstrap confidence interval included zero, H_7_ was not supported in the complete original theoretical model.

Although H_7_ was not supported in the complete original theoretical model, the nonsignificant direct paths corresponding to H_3_ and H_5_ suggested that a more parsimonious structure might warrant examination. Accordingly, a reduced model excluding these two paths was evaluated as a post hoc re-specification of the original model and was treated as exploratory rather than confirmatory. The results for this model are presented in Table 5.

Table 5 presents the direct path coefficients for the exploratory parsimonious model developed by excluding the X ⟶ M_2_ and M_1_ ⟶ Y paths that were not supported in the initial theoretical model. In the parsimonious model, the direct paths between social-emotional learning skills and emotional intelligence (β = 0.676, CR = 17.687, *p* < 0.001), emotional intelligence and emotion regulation (β = 0.476, CR = 10.439, *p* < 0.001), and emotion regulation and learning motivation (β = 0.133, CR = 2.528, *p* < 0.05) were positive and statistically significant. When the mediating variables were included in the model, the direct path between social-emotional learning skills and learning motivation was also positive and statistically significant (β = 0.207, CR = 3.929, *p* < 0.001). The bootstrap results for the serial indirect relationship in the exploratory parsimonious model are presented in Table 6.

**Figure 2:**
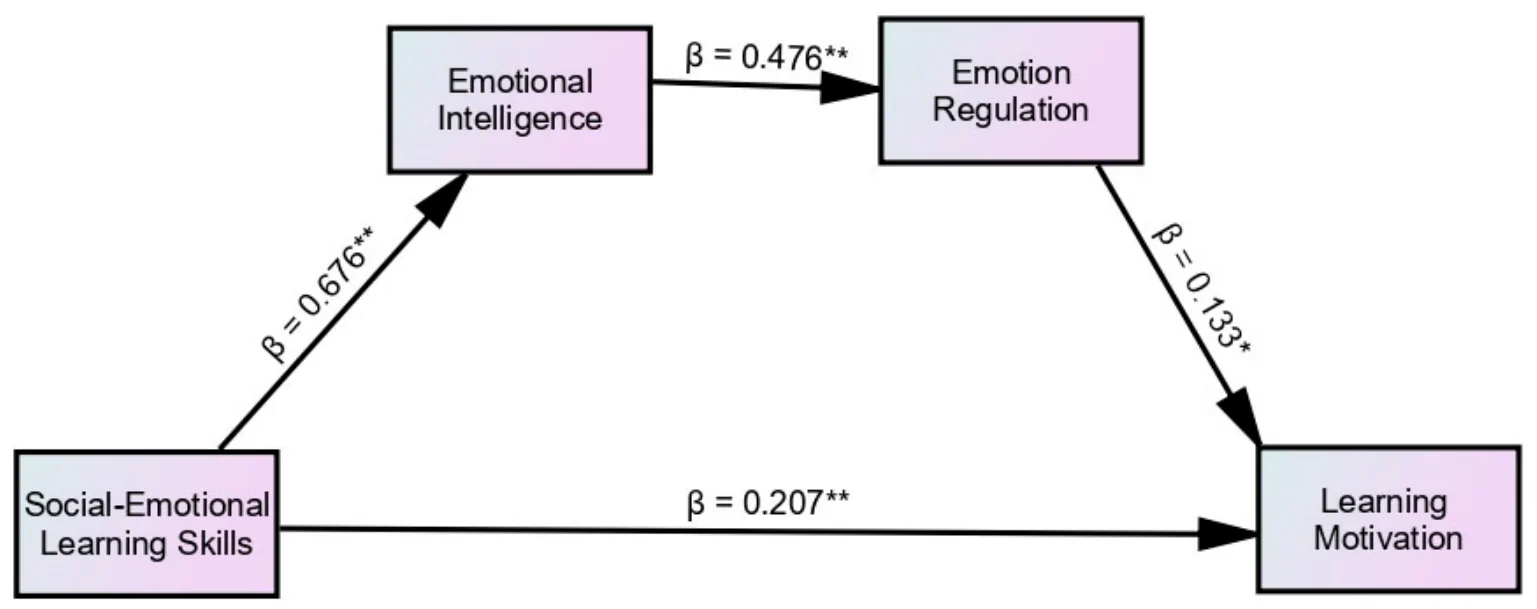
Standardized path coefficients for the exploratory parsimonious model. Note. Values represent standardized path coefficients. * *p* < 0.05, ** *p* < 0.001.

Table 6 presents the bootstrap results for the serial indirect relationship between social-emotional learning skills and learning motivation in the exploratory parsimonious model. The serial indirect relationship between social-emotional learning skills and learning motivation through emotional intelligence and emotion regulation was positive and statistically significant (β = 0.043, 95% CI [0.005, 0.082]). The fact that the bootstrap confidence interval did not include zero indicates that the serial indirect relationship in the exploratory parsimonious model was statistically significant. Moreover, the goodness-of-fit indices for the exploratory parsimonious model were within acceptable ranges (CMIN/DF = 0.72, RMR = 0.01, SRMR = 0.01, GFI = 0.99, NFI = 0.99, and RFI = 0.98). The standardized path coefficients for the exploratory parsimonious model are shown in Figure 2.

## 4. Discussion

The present study examined the relationships among university students’ SEL skills, emotional intelligence, emotion regulation, and learning motivation, with particular attention to the proposed serial indirect relationship involving emotional intelligence and emotion regulation. Of the six hypotheses concerning the total and direct relationships, H1, H2, H4, and H6 were supported, whereas H3 and H5 were not supported. In the complete original theoretical model, neither the total indirect relationship across all indirect paths nor the specific serial indirect relationship from SEL skills to learning motivation through emotional intelligence and emotion regulation was statistically significant. Accordingly, H7 was not supported in the original theoretical model. However, following a post hoc re-specification in which the two nonsignificant paths corresponding to H3 and H5 were removed, the exploratory parsimonious model revealed a statistically significant, although small, serial indirect relationship. Taken together, these results suggest that the relationships among the four constructs are more differentiated than initially proposed and that the sequential pattern identified in the exploratory model warrants further investigation rather than being interpreted as evidence of an established psychological mechanism.

The positive relationship between SEL skills and learning motivation supported H1. This finding is consistent with previous research linking social-emotional competencies with academic engagement, school connectedness, positive attitudes toward learning, and related academic outcomes (Cipriano et al., 2023; Durlak et al., 2011; Mahoney et al., 2018). The self-awareness and self-management components of SEL may be particularly relevant to students’ capacity to monitor their learning, establish goals, and regulate effort (Zins et al., 2004). From the perspective of self-determination theory, social-emotional competencies may also be related to experiences of autonomy, competence, and relatedness, which are closely connected with autonomous motivation (Bureau et al., 2022). Nevertheless, intervention evidence and the present correlational finding represent different forms of evidence. Previous meta-analytic studies largely concern outcomes related to participation in SEL interventions, whereas the present study examined individual differences in SEL skills. Accordingly, the current finding indicates that students reporting stronger SEL skills also tended to report greater learning motivation; it does not demonstrate that increasing SEL skills would necessarily produce an increase in learning motivation. This distinction is important when findings from intervention research are considered alongside individual-difference findings.

H2 was also supported, indicating a positive relationship between SEL skills and emotional intelligence. This finding is compatible with the CASEL framework because self-awareness and self-management include competencies involving the recognition and management of emotions (CASEL, 2020). Similar conceptual connections can be identified in the four-branch model of emotional intelligence, which encompasses perceiving, using, understanding, and managing emotional information (Mayer & Salovey, 1997; Mayer et al., 2016). Nevertheless, SEL and emotional intelligence should be understood as related but distinguishable constructs. SEL has a broader scope encompassing social awareness, relationship skills, and responsible decision-making in addition to emotional competencies, whereas emotional intelligence focuses more specifically on emotional information processing (Osher et al., 2016). Recent theoretical work also emphasizes that emotional intelligence as an ability does not automatically translate into emotionally intelligent behavior because motivation and contextual opportunities may influence whether emotional abilities are enacted (Levitats et al., 2025). The positive relationship observed in the present study therefore supports the theoretical relatedness of SEL skills and emotional intelligence without implying that they represent the same construct.

In contrast, H3 was not supported: SEL skills did not show a statistically significant unique direct relationship with emotion regulation in the initial theoretical model. This result requires cautious interpretation. Emotion regulation concerns more specific processes and strategies through which emotional responses are modified, and their functioning may vary according to the strategy and context involved (Gross, 2015; Gross & John, 2003). SEL, by comparison, encompasses a wider set of emotional, interpersonal, and decision-making competencies. Thus, conceptual overlap between SEL and emotion regulation does not necessarily imply that a unique direct relationship will remain when other closely related emotional characteristics are considered simultaneously. Previous theoretical work emphasizes the relevance of emotional awareness and emotional processing to regulatory functioning (Pekrun & Linnenbrink-Garcia, 2012; Zeidner et al., 2009). However, the nonsignificant H3 path should not be regarded as evidence that the relationship between SEL skills and emotion regulation necessarily operates through emotional intelligence. It indicates only that the proposed direct relationship was not statistically supported in the present sample.

The positive relationship between emotional intelligence and emotion regulation supported H4. This result is theoretically compatible with the four-branch model of emotional intelligence, in which the management of emotions represents a central domain of emotionally intelligent functioning (Mayer & Salovey, 1997). The capacity to perceive and understand emotional information may be related to how individuals select and implement regulatory responses in emotionally demanding situations (Mayer et al., 2016; Zeidner et al., 2009). Emotional intelligence has also been related to the management of stressful and anxiety-provoking emotional experiences (Salovey et al., 2002). At the same time, the conceptual distinction between the two constructs remains important. Emotional intelligence concerns a broader capacity for processing emotional information, whereas emotion regulation refers more specifically to the processes through which emotional responses are managed. The distinction between emotional ability and its behavioral enactment proposed by Levitats et al. (2025) further illustrates why emotional capacities and emotion-related behaviors should not be treated as equivalent.

H5, which proposed a direct positive relationship between emotional intelligence and learning motivation, was not supported. This finding does not necessarily contradict evidence linking emotional intelligence with academic functioning because academic performance, engagement, and learning motivation represent conceptually distinct outcomes. Meta-analytic evidence has demonstrated a relationship between emotional intelligence and academic performance (MacCann et al., 2020), while studies with university students have situated emotional intelligence, motivation, and academic functioning within broader relational networks. Chang and Tsai (2022), for example, examined emotional intelligence together with learning motivation and self-efficacy in relation to academic achievement, while Chin et al. (2025) identified a serial indirect relationship involving emotional intelligence, basic psychological needs, academic motivation, and student engagement. Recent theoretical work likewise suggests that possessing emotional ability alone may be insufficient to determine behavioral or motivational outcomes because motivation and contextual opportunities may shape how emotional abilities are expressed (Levitats et al., 2025). In the present study, however, the nonsignificant H5 path indicates only that emotional intelligence did not demonstrate a statistically significant unique direct relationship with learning motivation in the hypothesized model. It cannot by itself establish that emotion regulation explains this relationship.

H6 was supported, with emotion regulation showing a positive relationship with learning motivation. This result is consistent with recent studies indicating that emotion regulation strategies are related to academic motivation, approaches to learning, and academic functioning among university students (Rentzios et al., 2025; Wang & Wang, 2025). Chen et al. (2022) similarly reported relationships among emotion regulation, learning motivation, goal orientation, happiness, and psychological resilience. Research focusing on specific regulatory strategies also suggests that adaptive strategies such as cognitive reappraisal and maladaptive strategies such as suppression may show different relationships with motivational experiences (Liang & Mao, 2025). From the perspective of control-value theory, the regulation of academic emotions is closely connected with students’ motivational and self-regulatory experiences during learning (Pekrun & Linnenbrink-Garcia, 2012).

A central finding concerns the serial indirect relationship proposed in H7. In the initial theoretical model, the total indirect relationship across all indirect paths was not statistically significant (β = 0.072, 95% CI [−0.033, 0.176]). Because this estimate represented the combined indirect relationship rather than a separate estimate of the specific serial path, it should not be interpreted as a direct test of H7. The additional path-specific analysis showed that the specific serial indirect relationship from SEL skills to learning motivation through emotional intelligence and emotion regulation was also small and not statistically significant in the complete original theoretical model (β = 0.036, 95% CI [−0.0003, 0.0750]). Accordingly, H7 was not supported in the original theoretical model. Following examination of the initial model, the two nonsignificant paths corresponding to H3 and H5 were removed as part of a post hoc re-specification, and a more parsimonious model was additionally evaluated. In this exploratory parsimonious model, the serial indirect relationship between SEL skills and learning motivation through emotional intelligence and emotion regulation was statistically significant (β = 0.043, 95% CI [0.005, 0.082]). Accordingly, the exploratory result was consistent with the relational pattern proposed in H7, but it should not be regarded as confirmatory support for H7. This result is compatible with the possibility that the four constructs may form a sequential relational pattern. However, the finding requires considerable caution. The parsimonious model was specified after inspection of the results of the initial theoretical model and should therefore be treated as exploratory rather than confirmatory. Furthermore, the magnitude of the serial indirect relationship was small, and the lower confidence bound was close to zero. Statistical significance should consequently not be equated with substantial practical importance. Substantively, the small coefficient suggests that the sequential indirect relationship represents only a modest component of the broader association between SEL skills and learning motivation. Other direct, indirect, or unmeasured factors may therefore also contribute to the observed association between these constructs. Accordingly, the exploratory serial indirect relationship should not be interpreted as a dominant explanatory pathway or as providing a sufficient basis for practical intervention decisions. Finally, because the study employed a cross-sectional design, the ordering represented in the model cannot establish temporal precedence or causal sequencing.

The lack of support for H3, H5 and H7 in the complete original theoretical model should therefore not be interpreted as evidence that the corresponding relationships necessarily operate through the intermediate variables represented in the exploratory parsimonious model. Rather, the exploratory model identifies one statistical pattern that is compatible with the proposed ordering. This interpretation is broadly consistent with studies suggesting that emotional and motivational characteristics may be connected through multistep indirect relationships involving variables such as self-efficacy, social support, basic psychological needs, and academic motivation (Chin et al., 2025; Tang & He, 2023; Wang & Wang, 2025). However, the present findings do not establish a psychological mechanism or demonstrate that emotional intelligence temporally precedes emotion regulation. Replication in independent samples and longitudinal or experimental research will be necessary before stronger conclusions regarding the proposed sequential pattern can be drawn.

The findings may also be particularly relevant to teacher education given the composition of the sample. In the Turkish higher education context, students enrolled in faculties of arts and sciences may also pursue teaching careers by taking education-related elective courses during their undergraduate studies or by completing pedagogical formation education after graduation. Thus, although not all participants were formally enrolled in teacher education programs at the time of data collection, the sample included students from educational pathways that may lead to the teaching profession. Social-emotional characteristics may have distinctive importance in this context because students considering teaching careers may encounter emotional and interpersonal demands during their university education while preparing for a profession centered on sustained interaction with learners. The relationships observed among SEL skills, emotional intelligence, emotion regulation, and learning motivation therefore offer a potentially useful perspective for understanding the social-emotional dimensions of teacher preparation. Research in educational settings has likewise emphasized the broader relevance of emotion regulation and emotional support to educator functioning and well-being (Floman et al., 2024). Nevertheless, the present findings do not demonstrate that strengthening these characteristics would improve either learning motivation or future teaching competence. Instead, they indicate that these constructs warrant further examination in teacher education research, particularly through longitudinal and intervention studies addressing both pre-service learning and subsequent professional functioning.

## 5. Conclusions

This study examined the relationships among university students’ SEL skills, emotional intelligence, emotion regulation, and learning motivation, with particular attention to whether the observed data were consistent with a proposed serial indirect relationship involving emotional intelligence and emotion regulation. Of the six hypotheses concerning the total and direct relationships, H1, H2, H4, and H6 were supported, whereas H3 and H5 were not supported. SEL skills showed positive relationships with learning motivation and emotional intelligence; emotional intelligence showed a positive relationship with emotion regulation; and emotion regulation showed a positive relationship with learning motivation. In contrast, the proposed direct relationships between SEL skills and emotion regulation and between emotional intelligence and learning motivation were not statistically supported. The total indirect relationship across all indirect paths in the initial theoretical model was not statistically significant. More importantly, the specific serial indirect relationship from SEL skills to learning motivation through emotional intelligence and emotion regulation was also small and not statistically significant in the complete original theoretical model (β = 0.036, 95% CI [−0.0003, 0.0750]). Accordingly, H7 was not supported in the original theoretical model.

An exploratory parsimonious model was subsequently examined after the two nonsignificant paths corresponding to H3 and H5 were removed. This reduced model represented a post hoc re-specification of the original theoretical model and was therefore treated as exploratory rather than confirmatory. In this model, the serial indirect relationship between SEL skills and learning motivation through emotional intelligence and emotion regulation was statistically significant (β = 0.043, 95% CI [0.005, 0.082]). This exploratory finding is compatible with the hypothesized sequential ordering of the variables; however, it should not be interpreted as confirmatory support for H7 or as evidence of an established mediation mechanism. The magnitude of the serial indirect relationship was small, and the cross-sectional design does not establish temporal precedence or causal ordering. Accordingly, this exploratory pattern should primarily be regarded as a basis for further hypothesis testing. Thus, the exploratory finding was consistent with the pattern proposed in H7 but did not constitute confirmatory support for the original hypothesis.

The study contributes to the literature by examining SEL skills, emotional intelligence, emotion regulation, and learning motivation within the same integrated framework and by distinguishing among conceptually related but non-equivalent social-emotional constructs. The findings indicate that the relationships between social-emotional characteristics and learning motivation may be more differentiated than a simple sequential account would suggest. They also highlight the importance of considering both significant and nonsignificant paths when evaluating complex relational models rather than interpreting nonsignificant direct relationships as evidence of an indirect explanatory process.

The findings may also be relevant to curriculum development in higher education. The observed relationships between learning motivation and social-emotional characteristics suggest that curriculum developers may benefit from considering social-emotional dimensions alongside conventional disciplinary and academic learning outcomes during needs analysis, curriculum design, and evaluation. However, the present findings do not demonstrate that incorporating particular social-emotional components into curricula would improve learning motivation. Rather, they identify potential dimensions that warrant examination in future curriculum-development and intervention research.

This perspective may be particularly relevant to teacher education curricula given that the sample included students from educational pathways that may lead to the teaching profession. Social-emotional functioning may be important both to pre-service teachers’ experiences as university students and to their preparation for a profession involving sustained interpersonal interaction and emotional demands. The present study therefore provides a basis for further investigation of how SEL skills, emotional intelligence, emotion regulation, and learning motivation relate to the academic and professional development of prospective teachers. Stronger longitudinal and experimental evidence is needed before these relationships can be translated into specific recommendations for teacher education curricula.

## 6. Limitations and Recommendations for Future Research

The findings should be interpreted in light of several limitations. First, the study was conducted with voluntary participants from two faculties at a single university, which may have introduced self-selection bias and may limit the generalizability of the findings to other institutional and cultural contexts. Second, all constructs were assessed using self-report measures, which may be subject to response-related biases. Third, the cross-sectional design does not permit conclusions regarding temporal precedence or causality. Although previous evidence supports the use of the Social-Emotional Learning Skills Scale with university students, the instrument was originally developed for students in Grades 6–8, which should also be considered when interpreting the findings.

Future research should first examine whether the relational pattern identified in the present study can be replicated in independent samples. In particular, the serial indirect relationship identified in the exploratory parsimonious model should be specified a priori and tested in new samples before stronger conclusions are drawn regarding the proposed sequence among SEL skills, emotional intelligence, emotion regulation, and learning motivation. Longitudinal designs would be particularly valuable for examining whether the proposed ordering is supported over time. Studies involving participants from different universities, faculties, regions, and cultural contexts could also provide stronger evidence regarding the generalizability of the findings.

Future studies may extend the proposed model by examining additional variables that are theoretically relevant to students’ motivational experiences. Constructs such as self-efficacy, academic resilience, social support, instructional environment, and peer relationships may help clarify the broader relational network connecting social-emotional characteristics with learning motivation. Multiple data sources, including performance-based assessments, peer reports, and observational measures, could complement self-report instruments and reduce reliance on a single measurement method. Nevertheless, the ERQ was originally conceptualized as comprising two distinct dimensions, cognitive reappraisal and expressive suppression, which may have different relationships with academic and motivational outcomes. Therefore, combining the two dimensions into a single overall score may obscure strategy-specific associations. This issue should be considered when interpreting the present findings, and future studies should examine the two emotion-regulation strategies separately. Similarly, although the overall learning-motivation score provided a useful composite indicator for the present analyses, combining learning, performance-approach, and performance-avoidance goal orientations may obscure dimension-specific relationships. Future studies should therefore examine these motivational dimensions separately. In addition, the present study used observed-variable path analysis in AMOS, with each construct represented by its corresponding mean scale score. Although this approach enabled the simultaneous examination of direct and indirect relationships, it did not explicitly model measurement error or evaluate the measurement structure of the constructs within the structural model. This limitation may have affected the precision of the estimated relationships. Therefore, where sample size and psychometric conditions permit, future studies should test the proposed relationships using latent-variable structural equation modeling so that measurement error can be modeled explicitly and the measurement and structural models can be evaluated together.

Experimental and quasi-experimental research represents another important direction. The present correlational findings should not be interpreted as demonstrating that strengthening SEL skills, emotional intelligence, or emotion regulation would increase learning motivation. Future intervention studies could directly examine whether programs addressing these characteristics produce meaningful changes in motivational outcomes. The sequential pattern identified in the exploratory parsimonious model may provide one theoretically informed model for such research; however, alternative pathways and competing models should also be tested.

From a curriculum-development perspective, the findings may offer useful directions for higher education curricula. Curriculum developers could consider social-emotional characteristics as potential dimensions to be examined during needs analysis, curriculum design, learning-experience development, and curriculum evaluation rather than as predetermined elements that should automatically be incorporated into curricula. Pilot learning experiences involving self-awareness, emotional reflection, interpersonal interaction, or adaptive emotion regulation could be developed and empirically evaluated before broader curricular integration is considered. Future research could also compare whether social-emotional learning opportunities are more effective when embedded within disciplinary courses, organized as stand-alone curricular components, or connected with student-support services. Such evidence would help curriculum developers determine not only whether social-emotional dimensions are relevant to higher education curricula but also how and under what conditions they may be meaningfully incorporated.

Teacher education represents a particularly relevant context for this line of research. Future curriculum-development studies could examine whether dimensions such as self-awareness, emotional understanding, interpersonal skills, and adaptive emotion regulation warrant greater attention within teacher education curricula. These dimensions may be relevant both to pre-service teachers’ own motivational and academic experiences and to their preparation for the interpersonal and emotional demands of teaching. Rather than recommending their immediate inclusion as compulsory curricular components, the present findings support the use of needs analyses, pilot curriculum-development studies, and intervention research to determine whether and how social-emotional learning opportunities may contribute to teacher preparation. Future longitudinal studies could additionally examine whether social-emotional characteristics assessed during pre-service education are related to later outcomes such as classroom interaction, student support, professional motivation, emotion management, and teacher well-being.

Overall, the present findings should be regarded as providing directions for further curriculum development, teacher education, and intervention research rather than as prescriptive evidence for immediate curricular change. Replication of the exploratory parsimonious model, the use of latent-variable analyses, and stronger longitudinal and experimental evidence will be particularly important before specific curricular practices are recommended.

## Figures and Tables

**Table 1:** Descriptive statistics and normality results for the study variables.

Variable	M	SD	Skewness	Kurtosis	Shapiro–Wilk W	*p*	K–S D	*p*
SEL	3.08	0.38	−0.28	−0.08	0.99	<0.01	0.03	>0.05
EI	3.73	0.63	0.01	−0.22	0.98	<0.05	0.04	>0.05
ER	4.59	0.98	0.02	0.42	0.98	<0.05	0.06	<0.001
LM	2.12	0.38	0.06	−0.01	0.99	>0.05	0.05	<0.05

Note. N = 372. SEL = Social-Emotional Learning Skills; EI = Emotional Intelligence; ER = Emotion Regulation; LM = Learning Motivation; K–S = Kolmogorov–Smirnov test.

**Table 2:** Correlations among the principal study variables.

Variable	1	2	3	4
1. SEL	-			
2. EI	0.67 **	-		
3. ER	0.35 **	0.47 **	-	
4. LM	0.25 **	0.22 **	0.20 **	-

Note. N = 372. SEL = Social-Emotional Learning Skills; EI = Emotional Intelligence; ER = Emotion Regulation; LM = Learning Motivation. ** *p* < 0.001.

**Table 3:** Total relationship and direct path coefficients of the initial theoretical model.

Panel A. Total relationship model						
Hypothesis	Path	Relationship type	β	CR	*p*	Result
H_1_	X ⟶ Y	Total relationship	0.254	5.062	<0.001	Supported
Panel B. Initial theoretical serial indirect model						
Hypothesis	Path	Relationship type	β	CR	*p*	Result
H_2_	X ⟶ M_1_	Direct relationship	0.676	17.687	<0.001	Supported
H_3_	X ⟶ M_2_	Direct relationship	0.064	1.042	0.298	Not supported
H_4_	M_1_ ⟶ M_2_	Direct relationship	0.476	10.439	<0.001	Supported
H_5_	M_1_ ⟶ Y	Direct relationship	0.043	0.596	0.551	Not supported
H_6_	M_2_ ⟶ Y	Direct relationship	0.133	2.528	<0.05	Supported
-	X ⟶ Y (c′)	Direct relationship	0.182	2.687	<0.05	Significant

Note. The X ⟶ Y coefficient in Panel A was obtained from the total relationship model in which the mediating variables were not included. The coefficients in Panel B were obtained from the initial theoretical serial indirect model. X = Social-Emotional Learning Skills; M_1_ = Emotional Intelligence; M_2_ = Emotion Regulation; Y = Learning Motivation; β = Standardized Path Coefficient; CR = Critical Ratio; c′ = The direct relationship between X and Y when the mediating variables are included in the model.

**Table 4:** Bootstrap results for the initial theoretical model.

Relationship Type	Path	β	95% CI Lower	95% CI Upper	Result
Total indirect relationship	X ⟶ Y through all indirect paths	0.072	−0.033	0.176	Not significant
Specific serial indirect relationship	X → M_1_ → M_2_ → Y	0.036	−0.0003	0.0750	Not significant

Note. The total indirect relationship was estimated in AMOS using 5000 bootstrap resamples. The specific serial indirect relationship was estimated separately using PROCESS Macro for SPSS version 5.0 with 20,000 bootstrap resamples and is reported as a completely standardized indirect effect. X = Social-Emotional Learning Skills; M_1_ = Emotional Intelligence; M_2_ = Emotion Regulation; Y = Learning Motivation; CI = bootstrap confidence interval.

**Table 5:** Direct path coefficients of the exploratory parsimonious model.

Path	Relationship Type	β	CR	*p*	Result
X ⟶ M_1_	Direct relationship	0.676	17.687	<0.001	Significant
M_1_ ⟶ M_2_	Direct relationship	0.476	10.439	<0.001	Significant
M_2_ ⟶ Y	Direct relationship	0.133	2.528	<0.05	Significant
X ⟶ Y (c′)	Direct relationship	0.207	3.929	<0.001	Significant

Note. The X ⟶ M_2_ and M_1_ ⟶ Y paths, which were not statistically supported in the initial theoretical model, were excluded from the exploratory parsimonious model. X = Social-Emotional Learning Skills; M_1_ = Emotional Intelligence; M_2_ = Emotion Regulation; Y = Learning Motivation; β = Standardized Path Coefficient; CR = Critical Ratio; c′ = The direct path between X and Y when the mediating variables are included in the model.

**Table 6:** Bootstrap results for the exploratory parsimonious model.

Relationship Type	Path	β	95% CI Lower	95% CI Upper	Result
Serial indirect relationship	X ⟶ M_1_ ⟶ M_2_ ⟶ Y	0.043	0.005	0.082	Significant

## Data Availability

The data presented in this study are available upon request from the corresponding author due to ethical restrictions.
